# Moderately hypofractionated carbon ion radiotherapy for prostate cancer; a prospective observational study “GUNMA0702”

**DOI:** 10.1186/s12885-020-6570-8

**Published:** 2020-01-30

**Authors:** Hidemasa Kawamura, Nobuteru Kubo, Hiro Sato, Tatsuji Mizukami, Hiroyuki Katoh, Hitoshi Ishikawa, Tatsuya Ohno, Hiroshi Matsui, Kazuto Ito, Kazuhiro Suzuki, Takashi Nakano, Group for Genitourinary Tumors at Gunma University Heavy Ion Medical Center, Kazuhiro Suzuki, Kazuto Ito, Nobuaki Shimizu, Yutaka Takezawa, Junko Hirato, Hitoshi Ishikawa, Hiroshi Matsui, Hiroyuki Katoh, Tatsuya Ohno, Hidemasa Kawamura

**Affiliations:** 1 Gunma University Heavy Ion Medical Center, 3-39-22, Showa-machi, Maebashi, Gunma 371-8511 Japan; 2 Department of Radiation Oncology, Gunma University Graduate School of Medicine, Maebashi, Gunma Japan; 3 Ion-beam Radiation Oncology Center, Kanagawa Cancer Center, Yokohama, Kanagawa Japan; 4 Department of Radiation Oncology, Faculty of Medicine, University of Tsukuba, Tsukuba, Ibaraki Japan; 5 Department of Urology, Gunma University Graduate School of Medicine, Maebashi, Gunma Japan; 6 Institute for Preventive Medicine, Kurosawa Hospital, Maebashi, Gunma Japan

**Keywords:** Prostate cancer, Carbon ion radiotherapy, Prospective study

## Abstract

**Background:**

Carbon ion Radiotherapy for prostate cancer is widely used, however reports are limited from single institute or short follow up. We performed a prospective observational study (GUNMA0702) to evaluate the feasibility and efficacy of carbon ion radiotherapy for localized and locally advanced prostate cancer.

**Methods:**

Between June 2010 and August 2013, 304 patients with localized prostate cancer were treated, with a median follow-up duration of 60 months. All patients received carbon ion radiotherapy with 57.6 Gy (RBE) in 16 fractions over 4 weeks. Hormonal therapy was given according to the risk group. Toxicity was reported according to the Common Toxicity Criteria for Adverse Event, Version 4.0 by the National Cancer Institute.

**Results:**

The overall 5-year biochemical relapse-free rate was 92.7%, with rates of 91.7, 93.4, and 92.0% in low-risk, intermediate-risk, and high-risk patients, respectively. The 5-year local control and overall survival rates were 98.4 and 96.6%, respectively. Acute grade 3 or greater toxicity was not observed. Late grade 2 and grade 3 genitourinary and gastrointestinal toxicity rates were 9 and 0.3%, and 0.3, and 0%, respectively.

**Conclusions:**

The present protocol of carbon ion radiotherapy for prostate cancer provided low genitourinary and gastrointestinal toxicity with good biochemical control within 5 years.

**Trial registration:**

University Medical Information Network Clinical Trial Registry number: UMIN000003827.

## Background

The incidence of prostate cancer has increased in some European countries [1] and is increasing in Japan, Singapore, and China [2]. The number of patients diagnosed with localized prostate cancer is also increasing because of prostate-specific antigen (PSA) screening. Localized prostate cancer must be managed to maintain quality of life because they are not a life-threatening cancer in many cases. Radiation therapy could be an excellent treatment option for localized prostate cancer due to its effectiveness and low incidence of adverse events. Recently, the use of radiation for treating prostate cancer has increased by approximately 10% compared with previous Japanese studies [3].

Carbon ion radiotherapy (CIRT) has been used for localized and locally advanced prostate cancer. CIRT may represent an ideal treatment method for prostate cancer due to the unique physical and biologic advantages of carbon ion beams. The dose distribution of CIRT for prostate cancer is most advantageous for external beam irradiation techniques because of its superior dose characteristics [4]. Moreover, carbon ion beams have a high relative biological effectiveness (RBE), resulting from a high linear energy transfer, with their effect estimated to be approximately three times those of photons and protons [5, 6]. The first clinical trial of CIRT for prostate cancer was initiated at National Institute of Radiological Sciences (NIRS) in 1994. The efficacy and feasibility of CIRT for localized prostate cancer have been demonstrated through three phase I/II and two phase II clinical trials [7]. Appropriate dose fractionation schedules for CIRT and use of androgen deprivation therapy (ADT) according to tumor risk groups have also been reported. However, all previous reports of the use of this treatment are from the single institution; therefore, further trials by other institutions are required to validate these outcomes.

In 2009, Gunma University Heavy Ion Medical Center (GHMC) installed a new compact-sized accelerator and began providing CIRT since March 2010 [8]. Herein, we performed a prospective observational study (GUNMA0702) to confirm the efficacy and toxicity of CIRT for localized prostate cancer.

## Methods

### Patient eligibility

Patients aged between 20 and 80 years with histologically confirmed adenocarcinoma of the prostate staged as T1–T3N0M0 according to the International Union Against Cancer TNM classification (2002) were eligible for the present study. Assessments of Gleason Score (GS) were centrally reconfirmed for all tumors prior to study registration. Tumor stage was determined based on digital examination, trans-rectal ultrasound, computed tomography (CT), magnetic resonance imaging (MRI), and bone scanning. Exclusion criteria were history of pelvic RT or the presence of concurrent active malignancies or inflammatory disease. Patients who had received previous treatments for prostate cancer were also excluded.

### Central pathology review

All biopsy specimens were centrally re-evaluated by one pathologist at Gunma University Hospital. Tumor grade was assigned according to the modified Gleason grading system proposed by the International Society of Urological Pathology.

### Risk classification

Patients were stratified into three subgroups according to three major clinical risk factors of prostate cancer: T stage; initial prostate-specific antigen (iPSA) value; and GS. If patients with T1c–T2bN0M0 diseases had an iPSA <10 ng/ml and GS ≤ 6, they were allocated to the low-risk group. In contrast, patients with T3 or iPSA ≥20 ng/ml or GS ≥ 8 were assigned to the high-risk group. The remaining patients were classified as the intermediate-risk group.

### Androgen deprivation therapy

ADT consisted of medical castration by LH-RH agonists with or without anti-androgen and was administered according to our risk group criteria. For patients in the low-risk group, CIRT was performed without the use of ADT. Neo-adjuvant ADT was administered to patients in the intermediate- and high-risk groups for 5–8 months before the initiation of CIRT. Adjuvant ADT without anti-androgen was continued for high-risk patients only, and ADT was administered for a total of 24 months. Patients with T1c–T2b prostate cancer who had a GS of 7 (3 + 4) and an iPSA value less than 10 ng/ml were exceptionally regarded as having intermediate-risk but received CIRT without ADT.

### Carbon ion radiotherapy (CIRT)

A similar technique of CIRT for prostate cancer, which has been reported from NIRS, was used in the present study [7]. The feet of patients were positioned in a customized cradle (Moldcare; Alocare, Tokyo, Japan) and the pelvis was immobilized with a low-temperature thermoplastic sheet (Shellfitter; Kuraray, Co., Ltd., Osaka, Japan). At CT simulation, the bladder was filled with 100 mL sterilized saline and the rectum was emptied using an enema.

Treatment planning was performed using CT images of 2 mm thickness with fused MRI images with Xio-N (Elekta, Stockholm, Sweden and Mitsubishi Electric, Tokyo, Japan) [8]. The clinical target volume (CTV) included the prostate and the proximal seminal vesicles (SV). In T3b cases, we include the part of seminal vesicle as CTV where was involved by prostate cancer at diagnosis (pre neoadjuvant hormonal therapy) at least. The initial planning target volume (PTV1) was created by adding the anterior and lateral margins of 10 mm, cranial and caudal margins of 6 mm, and a posterior margin of 5 mm to the CTV, with lateral margins to the SV of 3 mm. According to the protocol from the NIRS, boost therapy was performed using the second PTV (PTV2), in which the posterior edge was set in front of the anterior wall of the rectum after the completion of nine fractions while the other margins remained the same as for PTV1 [9]. Each field was defined using spread-out Bragg peak and shaped by multi-leaf collimators and compensation bolus for each patient.

CIRT was performed at a total dose of 57.6 Gy (RBE) in 16 fractions over 4 weeks, with a fractional dose of 3.6 Gy (RBE) at four fractions a week. One field was used for each session, including one anterior field and a pair of lateral ports for PTV1 and another pair of lateral ports for PTV2. The bladder was also filled with 100 mL sterilized saline at each treatment session from the anterior direction. Patient positioning was three-dimensionally corrected using the same treatment couch used at the NIRS. All treatment plans were approved by the institutional conference prior to administering treatment.

### Assessment

Follow-up evaluations, including physical examination, blood testing for serum PSA, and urine screening, were performed at 3-month intervals. CT and MRI were performed once a year. Acute and late toxicities were evaluated using Common Terminology Criteria for Adverse Events version 4.0 (National Cancer Institute).

### Statistical considerations

The primary objective of the present study was bRFR at 5 years. Previous reports of bRFR at 5 years with X-ray IMRT were 91% in low-risk patients, 76% in intermediate-risk patients, and 68% in high-risk patients [10]. The reported bRFR with CIRT at the NIRS were 89% in low-risk patients, 97% in intermediate-risk patients, and 84% in high-risk patients [11–13]. Based on the assumption that the proportions of patients in low-risk, intermediate-risk, and high-risk groups are 6, 47, and 47%, respectively, 266 patients were planned to be enrolled at an overall type I error rate of α = 0.05 and power of 1 − β = 0.80.

Actuarial analysis was used to determine biochemical relapse-free survival by the Kaplan–Meier method. The OS, CSS, LCR, and bRFR were calculated from the CIRT start date. Biochemical failure was defined according to Phoenix criteria, as a rise of > 2.0 ng/mL above the PSA nadir [14].

## Results

### Patients accrual and registration

The present study was conducted from June 2010 to August 2013; a total of 309 patients were enrolled. The average monthly accrual for the study was 12.4 patients.

**Fig. 1:**
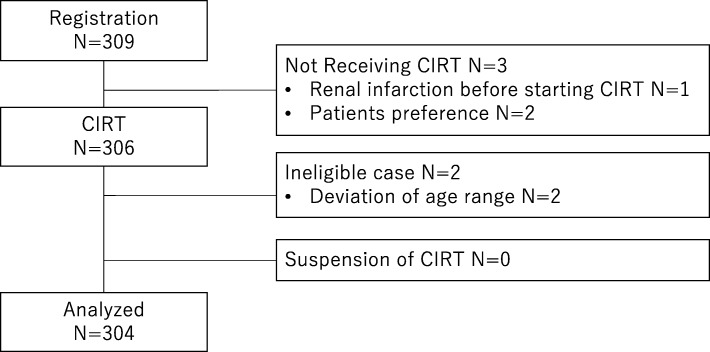
Flow diagram of analyzed patients

Two patients were judged as ineligible due to registration process errors, i.e., they were aged > 80 years at the time of registration. Three patients were registered but did not receive CIRT: two patients withdrew on their own after registration, and one patient developed renal infarction prior to the start of CIRT (Fig. 1). During the analysis of the 304 eligible patients, follow-up information was available for all patients. All patients in the present analysis had completed the scheduled CIRT. Three patients stopped ADT before the protocol regulated period due to unbearable toxicity and two patients received prolonged ADT with consent between patients and urologists.

### Patient characteristics

Table 1 lists the pretreatment characteristics of the 304 eligible patients. The median age of the patients was 66 years (range, 48–80 years). The majority of patients were stratified into the intermediate- (47%) or high-risk (48%) group, with only 5% of patients stratified into the low-risk group. Of these patients, 78% received ADT. The median follow-up period for surviving patients was 60.4 months.

**Table 1:** Patient Characteristics

Age (year)	66 (48–80)
T stage (UICC 2009)	
T1c	83 (27%)
T2a-b	86 (28%)
T2c	60 (20%)
T3a	69 (23%)
T3b	6 (2%)
Gleason Score	
6	20 (7%)
7	171 (56%)
3 + 4	97 (32%)
4 + 3	74 (24%)
8	36 (12%)
9	71 (23%)
10	6 (2%)
PSA (ng/ml)	
< 10	195 (64%)
10–20	75 (25%)
≧20	33 (11%)
Risk group	
Low	16 (5%)
Intermediate	142 (47%)
High	146 (48%)
ADT	
No	66 (22%)
Yes	238 (78%)

### Treatment results

**Fig. 2:**
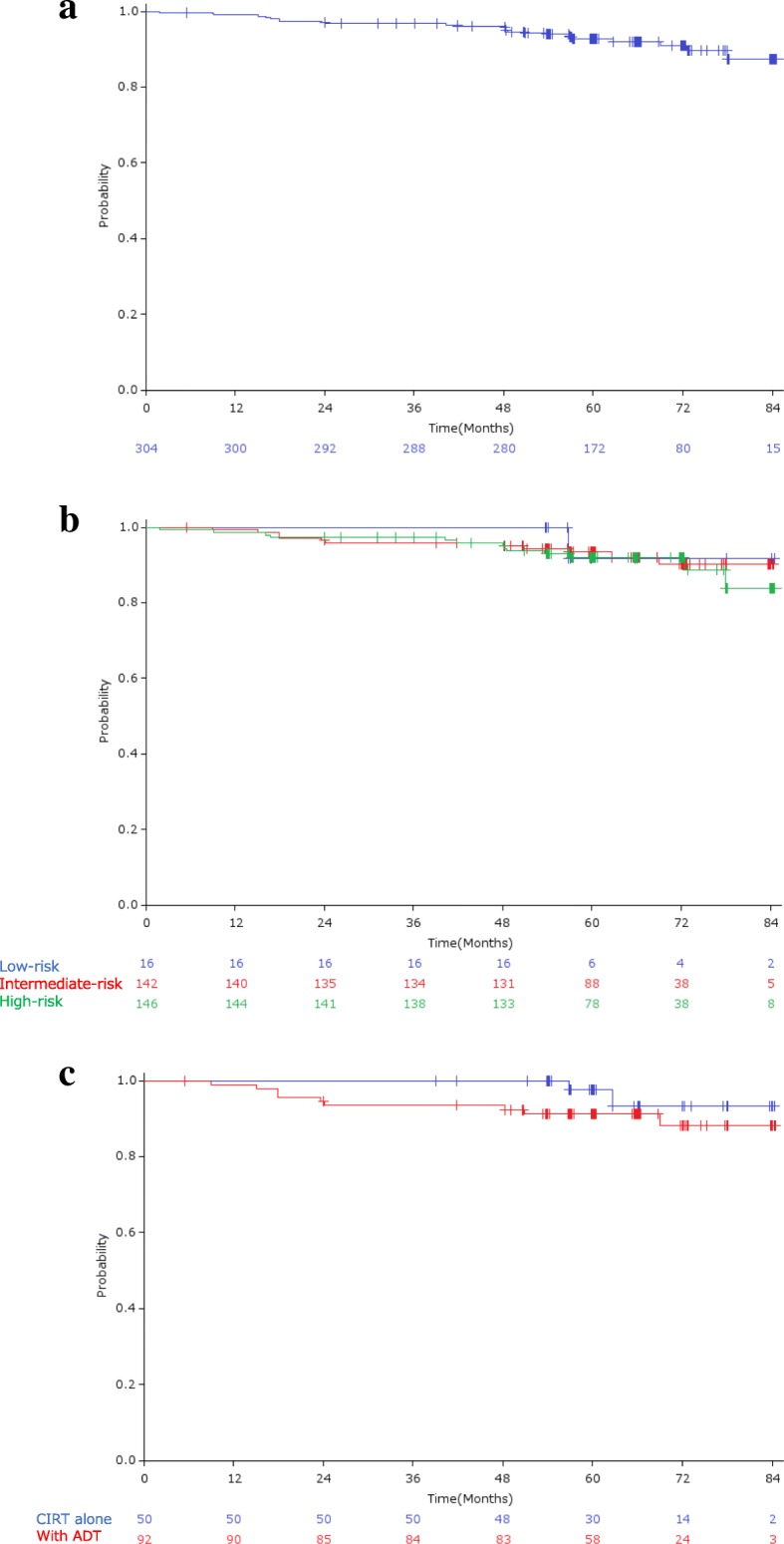
**a**) Biochemical relapse-free rate; **b**) Biochemical relapse-free rate by risk group; **c**) Biochemical relapse-free rate by use of ADT in intermediate-risk group patients

**Fig. 3:**
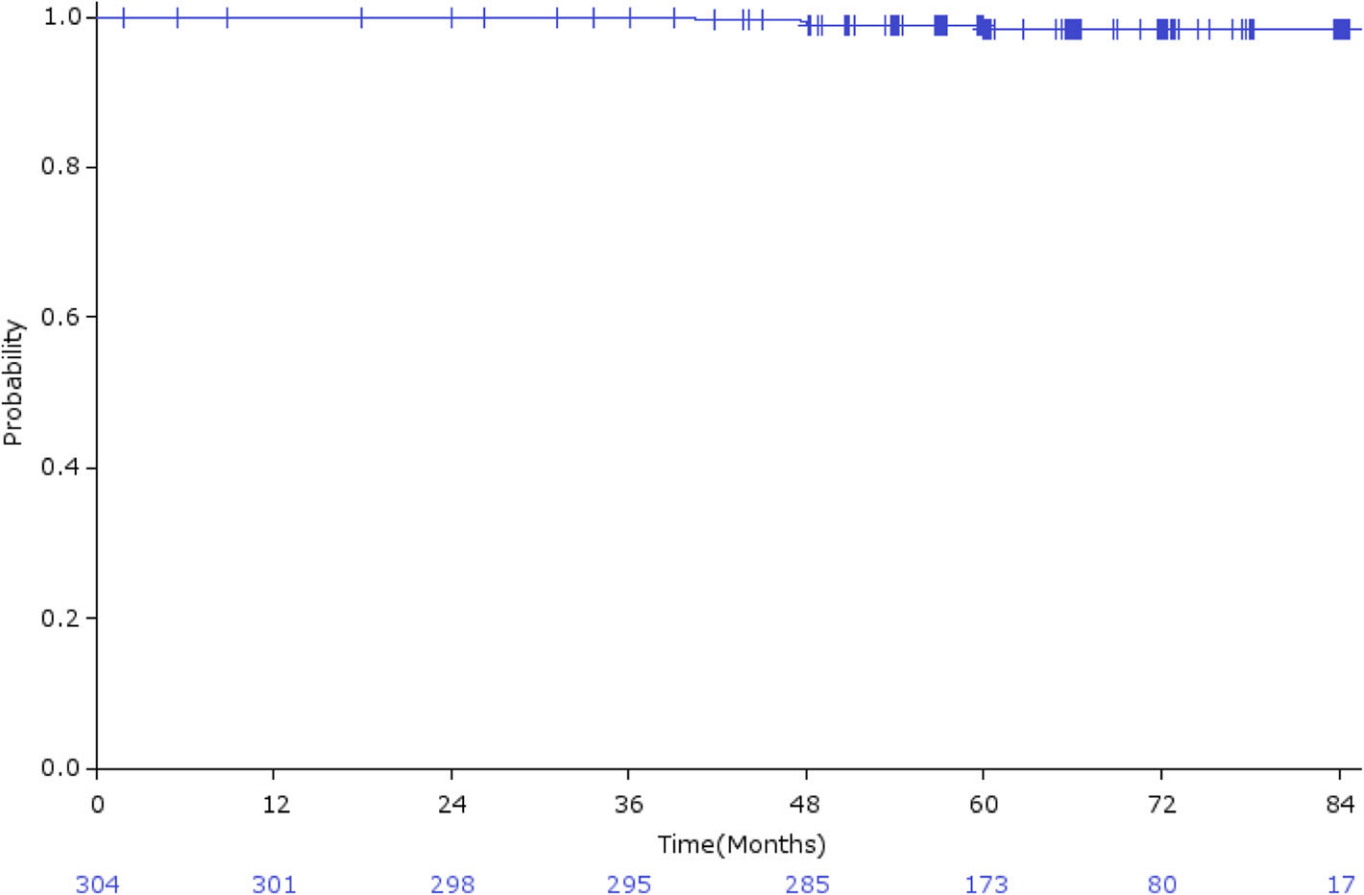
Local control rate

**Fig. 4:**
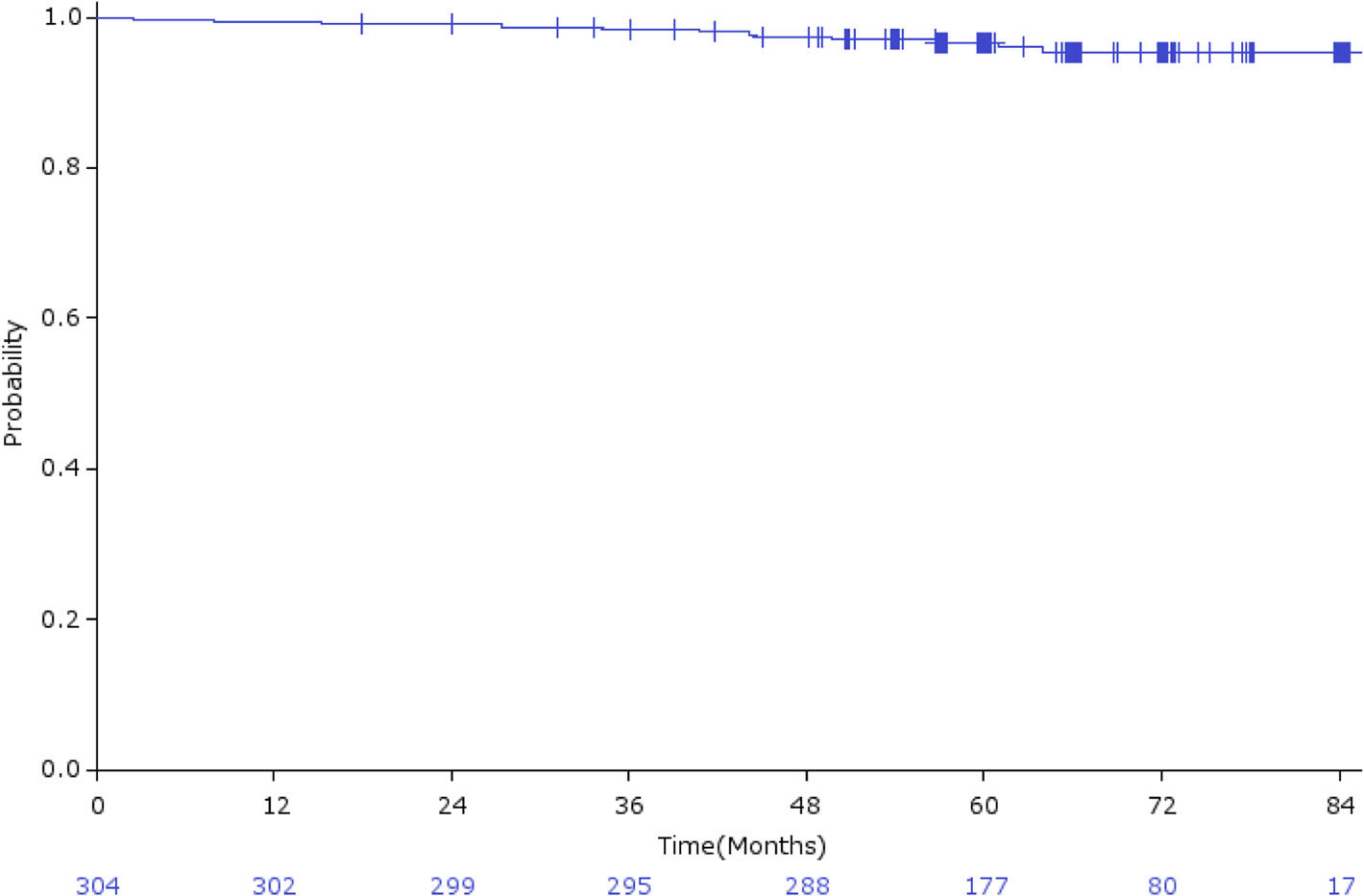
Overall survival rate

The overall 5-year bRFR was 92.7% (95% CI: 89.7–95.7%; Fig. 2a), and 91.7, 93.4, and 92.0% in low-risk, intermediate-risk, and high-risk patients, respectively (Fig. 2b). bRFR for intermediate-risk patients treated with CIRT alone and CIRT plus hormonal therapy were 97.5 and 91.1%, respectively (Fig. 2c). Local recurrence was observed in 4 (1.3%) patients, with a 5-year LCR of 98.4% (95% CI: 96.8–100%; Fig. 3). Distant metastasis was observed in 6 (2.0%) patients. Three patients (1.0%) died of prostate cancer and 9 (3.0%) died from other causes, with an OS rate of 96.6% (95% CI: 94.5–98.7%; Fig. 4).

### Toxicity

Table 2 shows acute and late toxicities following CIRT. Analysis of acute toxicities showed that the incidence of G2 and G3 genitourinary (GU) toxicities was 4.0, and 0%, respectively, and the incidence of G2–G3 gastrointestinal (GI) toxicities was 0%. Analysis of late toxicities revealed that the incidence of G2 and G3 GU toxicities was 9 and 0.3%, respectively, and the incidence of G2 and G3 GI toxicities was 0.3 and 0%, respectively. One patient experienced bladder bleeding requiring hyperbaric oxygen therapy, which was recorded as a late G3 GU toxicity. One patient experienced bleeding from the biopsied rectum site, which was recorded as a late G2 GI toxicity. GU and GI toxicities of grade 4 or greater were not observed.

**Table 2:** Acute and late toxicity

	Genitourinary toxicity				Gastrointestinal toxicity			
	G0	G1	G2	≧G3	G0	G1	G2	≧G3
Acute	131 (43%)	160 (53%)	13 (4%)	0 (0%)	300 (99%)	4 (1%)	0 (0%)	0 (0%)
Late Maximum	118 (39%)	156 (52%)	28 (9%)	1 (0.3%)	276 (91%)	26 (9%)	1 (0.3%)	0 (0%)
Late Last Follow up	236 (78%)	58 (19%)	9 (3%)	0 (0%)	302 (99.7%)	1 (0.3%)	0 (0%)	0 (0%)

## Discussion

The present study aimed to evaluate clinical outcomes of CIRT for prostate cancer at our institute with special regard to toxicities and determine the efficacy of our treatment protocol for localized prostate cancer. In particle therapy, beam characteristics and parameters differ by institutions because treatment planning systems and beam modification devices differ by institutions; therefore, the validation of treatment results is highly important to establish CIRT as a safe and effective treatment method. The CIRT dose fractionation was determined as 57.6 Gy (RBE) in 16 fractions over 4 weeks. This dose fractionation was recommended through prostate cancer treatment in NIRS [7]. The methods of patient fixation and basic design of PTV were similar to that used in NIRS.

Excellent treatment results with a low incidence of adverse events have been previously reported [7]. 1Ishikawa et al. reviewed the clinical outcomes of CIRT for prostate cancer over the last decade at NIRS, where CIRT was initiated for prostate cancer in 1995, and several phase I/II studies were performed to establish radiotherapy technique and determine the optimal radiation dose. Hypofractionation is also carried out; a total dose of 57.6 Gy (RBE) in 16 fractions was given over 4 weeks is almost equivalent based on the L/Q model (assuming α/β of prostate cancer cells is 2) to 63 Gy (RBE) in 20 fractions over 5 weeks. Furthermore, 51.6 Gy (RBE) in 12 fractions over 3 weeks is currently used [15]. They analyzed 927 patients, who have been followed up for at least 6 months. The 5-year biochemical relapse-free and LCRs were 90.6 and 98.3%, respectively. The 5-year biochemical relapse-free rates of the low-, intermediate-, and high-risk groups were 89.6, 96.8, and 88.4%, respectively.

Although excellent clinical results of CIRT for prostate cancer were demonstrated, they were reported only from one facility. Therefore, a multi-institutional retrospective study was conducted [16] to overcome this limitation. Data of 2157 patients enrolled in the prospective studies of 3 CIRT institutions were analyzed. The 5-year biochemical recurrence-free survival in low-, intermediate-, and high-risk patients was 92, 89, and 92%, respectively. Grade 3 or more toxicity was not observed. Thus, excellent treatment results with low toxicity were confirmed. However, this multi-institutional study was a retrospective study, and the observation period was only 29 months.

Our present analysis is the first prospective observational study conducted at a facility other than NIRS, with an observation period of 60 months. The 5-year bRFRs were 91.7, 93.4, and 92.0% in low-, intermediate-, and high-risk patients, respectively. Late grades 2 and 3 genitourinary and gastrointestinal toxicity rates were 9.0 and 0.3% and 0.3, and 0%, respectively. Therefore, this prospective study was the first to be conducted outside NIRS and to reproduce the previously reported treatment results, with a reasonably long follow up.

Recently, several clinical results of hypofractionated X-ray IMRT trials were reported. Randomized studies which compare hypofractionated schedule 60 Gy in 20 fractions (equivalent dose in 2 Gy fractions assuming α/β = 2 (EQD2) 75 Gy) with conventional fractionations (78 Gy in 39 fractions or 74 Gy in 37 fractions) showed that 5-year biochemical failure free rates of hypofractionated arms were 85–90.6% meeting noninferiority criteria without significant increase of late toxicity [17, 18]. Another study compared slightly higher dose 64.6 Gy in 19 fractions (EQD2 87.2 Gy) with conventional 78 Gy in 39 fractions and 5-year biochemical failure free rate was 80% and was not superior than conventional arm with slightly higher GI toxicity [19, 20]. Of course, it is difficult to compare these results with this current study because of differences in patients’ risk group, protocol design, ADT regimen and so on, our fractionation schedule 57.6 Gy (RBE) in 16 fractions (EQD2 81 Gy) CIRT shows reasonable 5-year biochemical relapse-free rate of 90.6% and promising GI toxicity rate with short treatment period.

Furthermore, diagnosis and treatment in the present study were homogenous, with the GS in all patients confirmed by a central pathological review. A recent study reported wide discordance in biopsy Gleason grading in one-third of all cases [21]. In the present study, CIRT was performed as planned in all patients, and hormonal therapy was planned in advance and performed with few protocol deviations. Of the 304 analyzed patients, only three patients suspended ADT. Therefore, the present study protocol demonstrated acceptable tolerability and quality.

In addition, we performed CIRT without ADT in favorable intermediate-risk patients. The definition of “favorable” intermediate-risk patients was not established at the time of this protocol design; therefore, this definition differs slightly from recent guidelines (e.g., the NCCN guideline). With our definition, favorable intermediate-risk patients demonstrated good PSA control without ADT. This result validates the efficacy of CIRT in “favorable” intermediate-risk patients with our definition.

The limitation of this study is that the median follow-up period of 60 months was relatively short in considering the long natural history of prostate cancer. Therefore, additional follow-up studies are required to evaluate the final outcomes of the present study.

## Conclusions

In conclusion, the present prospective study demonstrates excellent PSA control with a low incidence of severe toxicity following CIRT for prostate cancer. These outcomes are similar to those previously reported for CIRT, indicating reproducible efficacy. The present study may contribute to the establishment of CIRT as a one of the effective modality for the treatment of prostate cancer.

## Data Availability

The datasets generated and analyzed during the current study are not publicly available due to including personal information but are available from the corresponding author on reasonable request.

## Abbreviations

ADT: Androgen deprivation therapy
CIRT: Carbon ion radiotherapy
GHMC: Gunma University Heavy Ion Medical Center
GI: Gastrointestinal
GU: Genitourinary
NIRS: National Institute of Radiological Sciences
PSA: prostate-specific antigen
